# Prognoses of different pathological subtypes of colorectal cancer at different stages: A population-based retrospective cohort study

**DOI:** 10.1186/s12876-019-1083-0

**Published:** 2019-10-10

**Authors:** Xiaoli Wu, Han Lin, Shaotang Li

**Affiliations:** 1 Department of gastroenterology, the First Affiliated Hospital of Wenzhou Medical University, Wenzhou, Zhejiang People’s Republic of China; 2 Department of Central Laboratory, the First Affiliated Hospital of Wenzhou Medical University, Wenzhou, Zhejiang People’s Republic of China; 3 Department of Colorectal Surgery, the First Affiliated Hospital of Wenzhou Medical University, Wenzhou, Zhejiang People’s Republic of China

**Keywords:** Adenocarcinoma, Colorectal cancer, Mucinous adenocarcinoma, Prognosis, Signet ring cell carcinoma

## Abstract

**Background:**

Whether the prognoses of different pathological subtypes of colorectal cancer (CRC) at different stages are distinct is unclear.

**Methods:**

We extracted data on all cases of CRC from the Surveillance, Epidemiology, and End Results database between 2004 and 2015. The incidence of different pathological subtypes, clinical characteristics, and five-year overall survival (OS) and cause-specific survival (CSS) were analyzed.

**Results:**

A total of 384,996 cases diagnosed as adenocarcinoma (AC), mucinous adenocarcinoma (MAC), and signet ring cell carcinoma (SRCC) were included in this analysis. Compared with AC, MAC and SRCC were more likely to reach T4, N2, M1, stages III and IV, and grades III and IV, and patients were generally of a younger age (*P* < 0.001). Compared with those with AC, patients with MAC and SRCC showed poorer OS (50.6 and 26.8% vs. 60.2%, *P* < .001), with corresponding HR values of 1.238 (95% CI, 1.213–1.263, *P* < .001) and 1.592 (95% CI, 1.558–1.627, *P* < .001), respectively. The MAC and SRCC groups also showed poorer overall CCS (60.9 and 32.5% vs. 67.8%, *P* < .001), with corresponding HR values of 1.271 (95% CI, 1.242–1.302, *P* < .001) and 1.724 (95% CI, 1.685–1.765, *P* < .001), respectively. Compared with patients with AC, those with MAC showed poor OS at every stage and poor CSS at every stage except stage II (*P* < .05), while patients with SRCC revealed poor OS and CSS at every stage except stage 0 (P < .05).

**Conclusions:**

Patients of different pathological subtypes minimally differed at early stages. However, patients with AC have significantly better prognoses in advanced CRC (stages III and IV) than those with MAC or SRCC. Distinct treatment strategies should be applied depending on a particular histological subtype in advanced CRC.

## Background

CRC is the third most common malignancy and the second most common cause of death worldwide. About 1.4 million new cases are reported every year [1]. CRC is a significantly heterogeneous tumor with three major histological subtypes: AC, mucinous AC (MAC), and signet ring cell carcinoma (SRCC). Whereas typical ACs are the most common cancers of the colorectum, the two other pathological subtypes are rare and have characteristics distinct from those of AC, including a younger age of onset, more advanced stage, and increased likelihood of lymph node and peritoneal metastases upon presentation [2–6]. Although SRCC is widely believed to have poor prognosis [3, 5, 7], the prognosis of MAC remains unclear. A number of studies have demonstrated poorer outcomes in patients with MAC patients [8, 9], whereas other researchers have found different results [10, 11]. Several articles have demonstrated whether the prognoses of different histologic subtypes of colorectal cancer at different stages are distinct, but the results are unclear. Thus, the present study aimed to analyze the incidence of different pathological subtypes, clinical characteristics, and prognoses of different histologic subtypes of colorectal cancer at different stages.

## Methods

### Population selection

This study investigates the incidence rate, clinical characteristics, and oncological outcomes of patients with CRC. The data were extracted from the Surveillance, Epidemiology, and End Results (SEER) database and described in accordance with the items and codes documented by the North American Association of Central Cancer Registries [12]. Patients between 2004 and 2015 were extracted and coded in accordance with the year of diagnosis (Item 390). Tumor site and histology were coded in accordance with the criteria in the third edition of the International Classification of Diseases for Oncology [13]. Colorectal cancers included C18.0-cecum, C18.2-ascending colon, C18.3-hepatic flexure of the colon, C18.4-transverse colon, C18.5-splenic flexure of the colon, C18.6-descending colon, C18.7-sigmoid colon, C188-C189-large intestine, NOS, C19.9-rectosigmoid junction, and C20.9-rectum (Items 522 and 523). Patients who were diagnosed at autopsy or by death certificate only, who had another malignancy before CRC (Item 380), and who had no histologically confirmed cancer (Items 490 and 2180) were excluded from this study. This study was further stated patients with ACs, which were identified by histology codes 8140, 8144, 8210,8211, 8220, 8221,8255, 8260, 8261, 8262, 8263, mucinous 8480, mucin-producing adenocarcinoma 8481, and signet ring cell carcinoma 8490 (Item 522). Finally, this study was stated patients with clear stages as identified by the DERIVED AJCC-6 STAGE GRP (Item 3000).

### Statistical analysis

Data were analyzed using SAS statistical software (version 9.4; SAS Institute Inc.). Proportions were analyzed by the chi-squared test, and the correlations of each factor with OS and CSS were tested by logistic analysis. OS and CSS were also analyzed by the Kaplan–Meier method and Cox regression analysis.

## Results

### Study participants

**Fig. 1:**
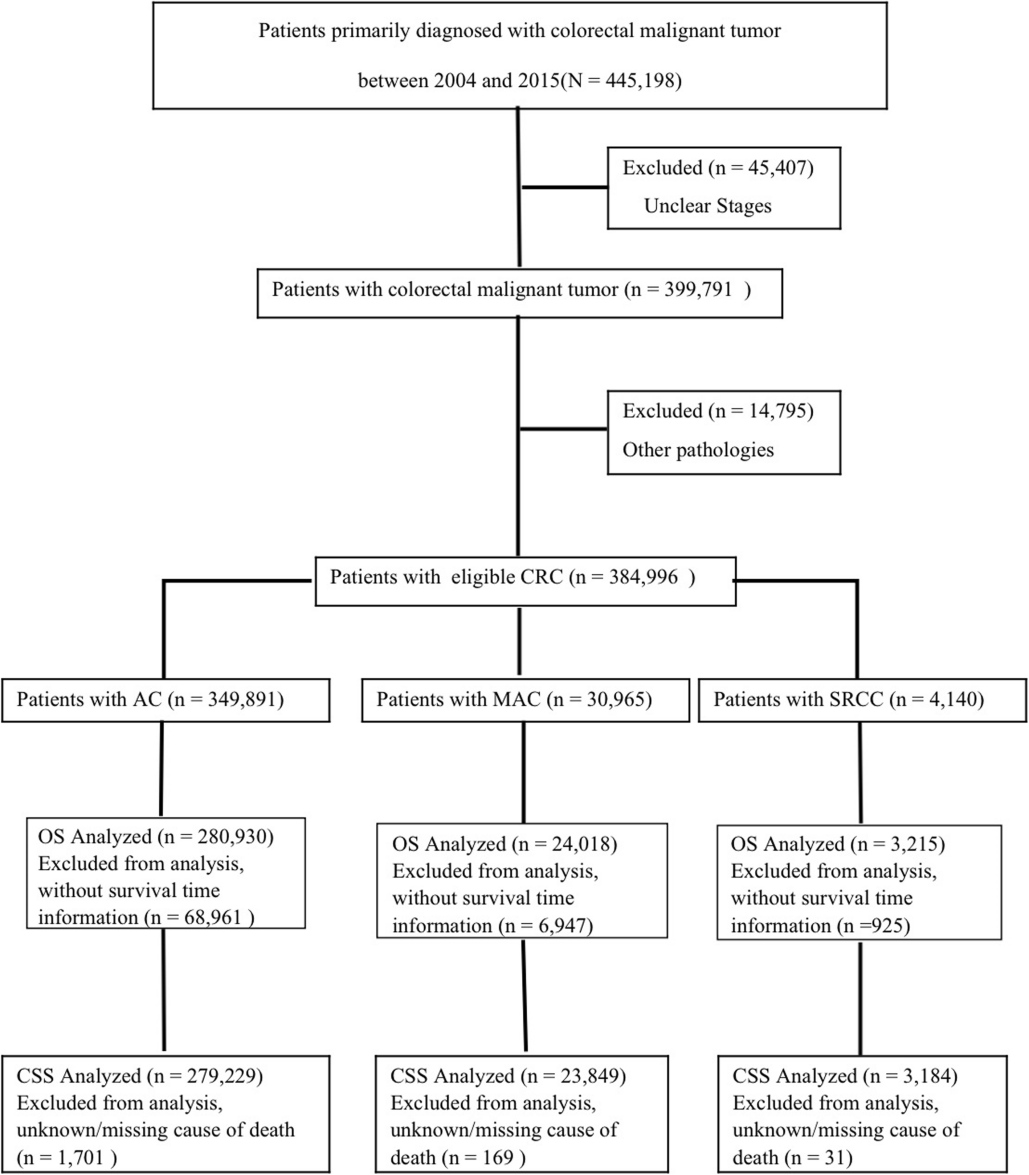
Selection process for patients in the cohort study

This study identified 445,198 patients who were diagnosed with colorectal malignant tumors between 2004 and 2015. After patients with unclear stage were excluded, a total of 399,791 patients were diagnosed with AC, MAC, SRCC, and other pathologies. The distribution of pathological subtypes was as follows: 87.5% (349,891 of 399,791) AC, 7.8% (30,965 of 399,791) MAC, 1.0% (4140 of 399,791) SRCC, and 3.7% (14,795 of 399,791) other pathologies. After patients with other pathologies were excluded, 384,996 patients remained in the cohort. Patients without survival time information were excluded from the OS analysis. Finally, 308,163 patients were retained in the cohort. After patients with unknown/missing cause of death were excluded from the CSS analysis, 306,262 patients remained in the study. Figure 1 lists the selection process for participants.

### Patients’ clinical characteristics

The patients’ clinical characteristics are listed in Table 1. In this study, approximately 90.1% (349,891) of the patients were diagnosed with AC. Compared with AC, MAC and SRCC were more likely to reach T4 (22.2 and 36.8% vs. 12.6%), N2 (19.7 and 41.0% vs.13.0%), M1 (20.3 and 38.5% vs.19.0%), stage III (32.0 and 39.5% vs. 25.7 25.7%), stage IV (20.3 and 38.5% vs. 19.0%), grade III (18.1 and 67.9% vs. 14.4%), and grade IV (2.8 and 11.2% vs. 1.7%), and patients with these cancers were of a relatively younger age (≤44 years, 5.9 and 11.6% vs. 5.0%), respectively (*P* < 0.001).

**Table 1:** Demographics and Clinical Characteristics of Patients with Colorectal Cancer in the Study

Variable	All Patients (*N* = 384,996)	Tumor Histology			*P* value
		AC (*N* = 349,891)	MAC (*N* = 30,965)	SRCC (*N* = 4140)	
Sex, No. (%)					< .001
Male	201,826 (52.4)	184,408 (52.7)	15,221 (49.2)	2197 (53.1)	
Female	183,170 (47.6)	165,483 (47.3)	15,744 (50.8)	1943 (46.9)	
Age, y, No. (%)					< .001
≤ 44 y	19,932 (5.2)	17,632 (5.0)	1819 (5.9)	481 (11.6)	
45-59y	91,886 (23.9)	84,785 (24.2)	6151 (19.9)	950 (22.9)	
60-74y	141,249 (36.7)	129,226 (36.9)	10,646 (34.4)	1377 (33.3)	
≥ 75y	131,929 (34.3)	118,248 (33.8)	12,349 (39.9)	1332 (32.2)	
Marital status, No. (%)					< .001
Unmarried	205,102 (53.3)	186,964 (53.4)	15,941 (51.5)	2197 (53.1)	
Married	160,284 (41.6)	144,728 (41.4)	13,778 (44.5)	1778 (42.9)	
Unknown	19,610 (5.1)	18,199 (5.2)	1246 (4.0)	165 (4.0)	
T, No. (%)					< .001
T0	567 (0.1)	499 (0.1)	60 (0.2)	8 (0.2)	
Tis	14,784 (3.8)	14,650 (4.2)	108 (0.3)	26 (0.6)	
T1	67,426 (17.5)	65,451 (18.7)	1692 (5.5)	283 (6.8)	
T2	50,455 (13.1)	46,734 (13.4)	3550 (11.5)	171 (4.1)	
T3	180,679 (46.9)	161,305 (46.1)	17,606 (56.9)	1768 (42.7)	
T4	52,617 (13.7)	44,210 (12.6)	6884 (22.2)	1523 (36.8)	
Tx	18,468 (4.8)	17,042 (4.9)	1065 (3.4)	361 (8.7)	
N, No. (%)					< .001
N0	230,313 (59.8)	212,807 (60.8)	16,251 (52.5)	1255 (30.3)	
N1	89,719 (23.3)	80,920 (23.1)	7874 (25.4)	925 (22.3)	
N2	53,294 (13.8)	45,509 (13.0)	6087 (19.7)	1698 (41.0)	
NX	11,670 (3.0)	10,655 (3.0)	753 (2.4)	262 (6.3)	
M, No. (%)					< .001
M0	310,099 (80.5)	282,887 (80.9)	24,669 (79.7)	2543 (61.4)	
M1	74,512 (19.4)	66,626 (19.0)	6291 (20.3)	1595 (38.5)	
MX	385 (0.1)	378 (0.1)	5 (0.0)	2 (0.0)	
Stage, No. (%)					< .001
0	14,784 (3.8)	14,650 (4.2)	108 (0.3)	26 (0.6)	
I	95,327 (24.8)	91,062 (26.0)	4007 (12.9)	258 (6.2)	
II	98,990 (25.7)	87,722 (25.1)	10,643 (34.4)	625 (15.1)	
III	101,383 (26.3)	89,831 (25.7)	9916 (32.0)	1636 (39.5)	
IV	74,512 (19.4)	66,626 (19.0)	6291 (20.3)	1595 (38.5)	
Grade, No. (%)					< .001
I	32,258 (8.4)	29,373 (8.4)	2861 (9.2)	24 (0.6)	
II	244,735 (63.6)	226,222 (64.7)	18,318 (59.2)	195 (4.7)	
III	58,637 (15.2)	50,223 (14.4)	5603 (18.1)	2811 (67.9)	
IV	7371 (1.9)	6053 (1.7)	853 (2.8)	465 (11.2)	
Unknown	41,995 (10.9)	38,020 (10.9)	3330 (10.8)	645 (15.6)	

### Prognostic factors

The factors correlated with prognosis (OS and CSS) are listed in Table 2. Logistic analysis demonstrated the following factors associated with poor prognosis: male sex, old age (≥60 years), unmarried status, histopathology grades 3 and 4, MAC and SRCC, and stages III and IV (*P* < 0.001). Among these factors, stage classification and pathological subtype were the two most significantly associated with disease prognosis.

**Table 2:** Factors Associated with the Survival of Patients with CRC

Covariate	5-y Overall Survival			5-y Cancer-Specific Survival		
	Total No. (OS, %)	Logistic Analysis		Total No. (CSS, %)	Logistic Analysis	
		Hazard Ratio (95% CI)	P Value		Hazard Ratio (95% CI)	*P* Value
Sex						
Male	161,545 (56.7)	1 [Reference]		160,455 (66.6)	1 [Reference]	
Female	146,618 (57.6)	0.964 (0.950–0.978)	< .001	145,807 (67.2)	0.973 (0.959–0.988)	< .001
Age, y						
≤ 44 y	18,591 (65.2)	1 [Reference]		18,463 (67.1)	1 [Reference]	
45-59y	82,523 (67.1)	0.919 (0.888–0.950)	< .001	82,076 (70.6)	0.849 (0.821–0.879)	< .001
60-74y	114,178 (61.8)	1.158 (1.121–1.196)	< .001	113,505 (69.7)	0.887 (0.858–0.917)	< .001
≥ 75y	92,871 (41.5)	2.641 (2.556–2.729)	< .001	92,218 (60.1)	1.354 (1.310–1.400)	< .001
Marital status						
Married	165,165 (62.8)	1 [Reference]		164,265 (70.8)	1 [Reference]	
Unmarried	127,587 (49.1)	1.750 (1.724–1.776)	< .001	126,716 (61.1)	1.544 (1.520–1.568)	< .001
Unknown	15,411 (61.7)	1.048 (1.013–1.084)	.007	15,281 (72.1)	0.938 (0.904–0.974)	< .001
0	11,192 (80.0)	1 [Reference]		11,142 (93.6)	1 [Reference]	
I	72,764 (77.7)	1.148 (1.093–1.206)	< .001	72,462 (91.4)	1.376 (1.270–1.491)	< .001
II	78,593 (68.3)	1.857 (1.768–1.949)	< .001	78,201 (81.6)	3.298 (3.050–3.565)	< .001
III	83,499 (59.4)	2.734 (2.605–2.869)	< .001	83,024 (67.9)	6.914 (6.400–7.469)	< .001
IV	62,115 (10.6)	33.736 (31.998–35.568)	< .001	61,433 (12.1)	106.242 (98.109–115.050)	< .001
Histology						
AC	280,930 (58.0)	1 [Reference]		279,229 (67.8)	1 [Reference]	
MAC	24,018 (50.7)	1.343 (1.308–1.379)	< .001	23,849 (60.9)	1.352 (1.316–1.389)	< .001
SRCC	3215 (26.8)	3.772 (3.487–4.079)	< .001	3184 (32.5)	4.373 (4.059–4.712)	< .001
Grade						
Grade I	25,618 (69.4)	1 [Reference]		25,478 (81.1)	1 [Reference]	
Grade II	197,250 (60.7)	1.468 (1.428–1.510)	< .001	196,101 (70.9)	1.761 (1.704–1.820)	< .001
Grade III	46,599 (44.4)	2.840 (2.750–2.933)	< .001	46,277 (52.7)	3.851 (3.714–3.994)	< .001
Grade IV	5770 (44.7)	2.806 (2.647–2.974)	< .001	5741 (53.3)	3.760 (3.539–3.995)	< .001
Unknown	32,926 (46.2)	2.641 (2.552–2.733)	< .001	32,665 (54.6)	3.568 (3.434–3.707)	< .001

### Pathological subtypes and prognoses at every stage

**Fig. 2:**
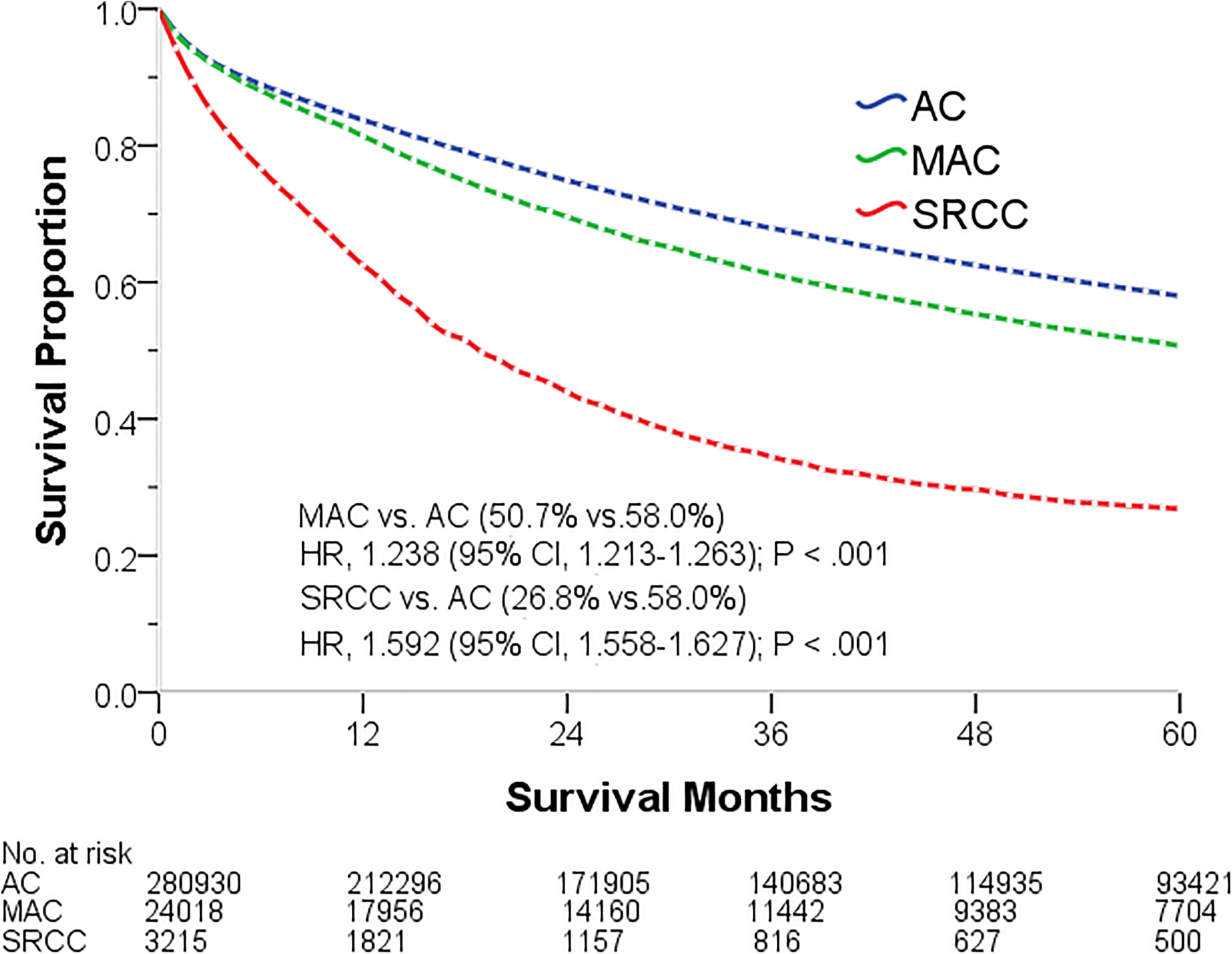
Five-year overall survival of patients of different pathological subtypes

**Fig. 3:**
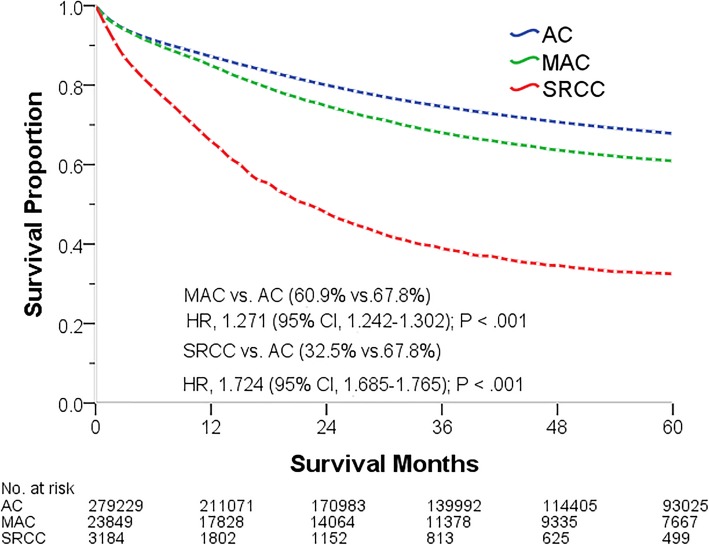
Five-year cause-specific survival of patients of different pathological subtypes

This study performed exploratory analyses to demonstrate the associations of the pathological subtype with OS and CSS. The pathological subtypes were found to be correlated with the prognosis. Compared with patients with AC at all stages (0–IV), those with MAC and SRCC displayed a poorer OS (50.6 and 26.8% vs. 60.2%, *P* < .001), with corresponding HR values of 1.238 (95% CI, 1.213–1.263, *P* < .001) and 1.592 (95% CI, 1.558–1.627, *P* < .001), respectively. Similarly, compared with patients with AC, those with MAC and SRCC showed a poor CCS (60.9 and 32.5% vs. 67.8%, *P* < .001), with corresponding HR values of 1.271 (95% CI, 1.242–1.302, *P* < .001) and 1.724 (95% CI, 1.685–1.765, *P* < .001), respectively (Tables 3, 4). Patients with MAC and SRCC generally showed poor OS and CCS (Figs. 2, 3), although different pathologies resulted in significantly different prognoses (logrank [Mantel–Cox], *P* < .001). The median OS and CSS of patients with SRCC were 19 and 23 months, respectively. We further analyzed the correlation between pathological subtype and OS and CCS values at every stage. Cox regression analysis demonstrated that, compared with patients with AC, those with MAC have poorer OS at every stage and poorer CSS at every stage except at stage II (*P* < .05, Tables 3, 4). Patients with SRCC had poorer OS and CSS at every stage except stage 0 (*P* < .05, Tables 3, 4).

**Table 3:** Histology Correlated With Overall Survival among 308,163 Patients with Colorectal Cancer

Stage	Histology			Cox Regression			
	AC Total No. (5-y OS, %)	MAC Total No. (5-y OS, %)	SRCC Total No. (5-y OS, %)	MAC vs. AC HR (95) CI%	*P* Value	SRCC vs. AC HR (95) CI%	P Value
0-IV	280,930 (58.0)	24,018 (50.7)	3215 (26.8)	1.238 (1.213–1.263)	< .001	1.592 (1.558–1.627)	< .001
0	11,113 (80.1)	66 (69.1)	13 (83.1)	1.656 (1.054–2.602)	.029	0.954 (0.477–1.909)	.895
I	69,751 (77.9)	2848 (72.7)	165 (63.4)	1.268 (1.172–1.371)	< .001	1.387 (1.207–1.593)	< .001
II	70,050 (68.6)	8075 (66.6)	468 (56.0)	1.072 (1.029–1.122)	.001	1.279 (1.188–1.377)	< .001
III	74,341 (60.6)	7863 (52.6)	1295 (34.6)	1.297 (1.250–346)	< .001	1.519 (1.464–1.576)	< .001
IV	55,675 (10.8)	5166 (10.0)	1274 (2.5)	1.053 (1.020–1.087)	.001	1.245 (1.208–1.282)	< .001

**Table 4:** Histology Correlated With Cause-Specific Survival among 306,262 Patients with Colorectal Cancer

Stage	Histology			Cox Regression			
	AC Total No. (5-y CSS, %)	MAC Total No. (5-y CSS, %)	SRCC Total No. (5-y CSS, %)	MAC vs. AC HR (95) CI%	P Value	SRCC vs. AC HR (95) CI%	P Value
0-IV	279,229 (67.8)	23,849 (60.9)	3184 (32.5)	1.271 (1.242–1.302)	< .001	1.724 (1.685–1.765)	< .001
0	11,064 (93.7)	65 (83.4)	13 (83.1)	2.592 (1.342–5.007)	.005	1.726 (0.862–3.455)	.123
I	69,464 (91.4)	2833 (90.1)	165 (74.6)	1.174 (1.027–1.341)	.018	1.828 (1.540–2.169)	< .001
II	69,705 (81.7)	8031 (81.6)	465 (70.3)	1.016 (0.957–1.079)	.604	1.372 (1.251–1.506)	.011
III	73,923 (69.1)	7816 (61.1)	1285 (41.1)	1.356 (1.300–1.415)	< .001	1.630 (1.565–1.697)	< .001
IV	55,073 (12.4)	5104 (11.4)	1256 (3.2)	2.218 (2.146–2.293)	< .001	1.734 (1.681–1.788)	< .001

## Discussion

Previous studies on the prognostic effects of different pathological subtypes in CRC have yielded conflicting results [8–11]. Therefore, to date, no clinical guidelines have yet been established for the different treatment methods for CRC of different pathological subtypes. For this reason, we conducted a population-based study to analyze the prognoses of different pathological subtypes in patients with CRC. We found that MAC and SRCC were associated with various clinicopathological characteristics, such as a younger age, poorer grade of differentiation, easier metastasis, and advanced stage. These findings are consistent with those of previous researchers [2–4, 6, 7]. We also found that the prognoses statistically differed among patients with different pathological subtypes in terms of overall outcome; specifically, the pathological subtypes were correlated with the prognosis. Patients with AC had the best prognoses, whereas those with SRCC exhibited the poorest prognoses. The median OS and CSS of patients with SRCC were only 19 and 23 months, respectively. To explore the correlations between the prognoses and different pathological subtypes of patients with CRC at every stage, stage-specific Cox regression analysis was performed. We found that patients of different pathological subtypes were only minimally different, and no statistical difference at the early stages of the disease (i.e., no lymph node metastasis) was observed. Thus, patients with SRCC had OS and CSS similar to those with AC at stage 0 (*P* = 0.895 and *P* = 0.123, respectively), and patients with MAC had CSS similar to those with AC at stage II (*P* = 0.604). A small number of recent studies demonstrated that SRCC does not negatively affect survival in stage I and II colorectal tumors [14].

In contrast to findings among early-stage CRC, patients with MAC and SRCC had poor OS and CSS in advanced CRC (stages III and IV). The key pathological difference among the three subtypes is that both MAC and SRCC produce large amounts of mucin. The World Health Organization defines MAC as a carcinoma conformed by > 50% of extracellular mucin pools that contain malignant epithelial or.

individual tumor cells including signet-ring cells (SRC); it defines SRCC as a carcinoma conformed by > 50% of SRC. A unique pathologic feature of SRCC is that SRC have intracytoplasmic mucin vacuoles that displace the nuclei to the periphery.

Previous researchers have demonstrated the importance of mucin in prognosis [15, 16]. One study enrolled patients with CRC of different stages and receiving adjuvant chemotherapy via the FOLFOX regimen [15]. Another recent study enrolled only patients with stage III CRC who were also receiving adjuvant chemotherapy via the FOLFOX regimen [16]. The results of these studies showed that patients with MAC have poorer prognoses than those without mucin. This research reveals that patients with MAC have significantly poorer prognoses than those with AC. The poor prognoses for patients with MAC and SRCC may be due to aggressive infiltrating tumor growth, which promotes higher rates of lymphovascular invasion [17].

We found that patients with MAC and SRCC experienced lymph node metastasis more frequently than those with MAC (47.5 and 69.7% vs. 39.2%). Other studies investigating the molecular characteristics of SRCC have demonstrated that most SRCC cases feature variable molecular alterations, including highly microsatellite instability, a high CpG island methylator phenotype, and high frequency of BRAF V600E mutation [18–22]. The poor prognoses of patients with SRCC may also be explained by the low suitability of the existing standard treatment approach for these patients [23]. Improving the treatment approach requires special schemes based on the genetic background of the disease.

### Limitations and strengths

To the best of our knowledge, this work is the most comprehensive study exploring the correlation between different pathological subtypes and prognoses of patients with CRC. This study presents a number of strengths. First, our data come from naturally registered patients, who are highly popular, which means our data are credible. Second, our data spanned a period of over 10 years and included more than 384,000 patients. Larger sample sizes tend to be more reliable than smaller ones. Third, we used different analytical methods to prove the consistency and reliability of this results. Nevertheless, this research also presents a number of limitations. In particular, data on treatment approaches, performance status, and molecular features (e.g., MSI status and BRAF mutation) were unavailable in the SEER database.

## Conclusions

This population-based cohort study on patients with CRC of different pathological subtypes provided compelling evidence that different pathological subtypes are only minimally different at early stages. However, they were significantly different prognosis, and patients with MAC and SRCC have poorer OS and CCS, which are mainly in advanced CRC (stages III and IV). Therefore, different treatment strategies specific for a particular histological subset should be applied in advanced CRC.

## Data Availability

The data used and/or analyzed during the study are available from the corresponding author on reasonable request.

## Abbreviations

AC: Adenocarcinoma
CRC: Colorectal cancer
CSS: Cause-specific survival
ICDO: International Classification of Diseases for Oncology
MAC: Mucinous adenocarcinoma
NAACCR: North American Association of Central Cancer Registries
OS: Overall Survival
SEER: Surveillance, epidemiology, and end results
SRCC: Signet ring cell carcinoma
